# Case report: Acute toxic myocardial damage caused by 5-fluorouracil—from enigma to success

**DOI:** 10.3389/fcvm.2022.991886

**Published:** 2022-10-18

**Authors:** Ratko Lasica, Jelena Spasic, Lazar Djukanovic, Danijela Trifunovic-Zamaklar, Dejan Orlic, Olga Nedeljkovic-Arsenovic, Milika Asanin

**Affiliations:** ^1^Department of Cardiology, Emergency Center, University Clinical Center of Serbia, Belgrade, Serbia; ^2^Institute for Oncology and Radiology of Serbia, Belgrade, Serbia; ^3^Department of Cardiology, University Clinical Center of Serbia, Belgrade, Serbia; ^4^Center for Radiology and Magnetic Resonance, University Clinical Center of Serbia, Belgrade, Serbia

**Keywords:** cancer, 5-fluorouracil, toxic myocardial damage, cardiomyopathy, acute coronary syndrome

## Abstract

Considering the pandemic of both cardiovascular diseases and oncological diseases, there is an increasing need for the use of chemotherapy, which through various pathophysiological mechanisms leads to damage to heart function. Cardio toxicity of chemotherapy drugs can manifest itself in a variety of clinical manifestations, which is why establishing a valid diagnosis is a real mystery for clinicians. Acute systolic heart failure (AHF) due to the use of 5-fluorouracil (5-FU) is a rare occurrence if it is not associated with myocardial infarction, myocarditis or Takotsubo cardiomyopathy. Therefore, we decided to present a case of an 52-year-old male who was diagnosed with stage IV RAS wild-type adenocarcinoma of the rectum and in whom the direct toxic effect 5-FU is the main reason for the appearance of toxic cardiomyopathy.

## Introduction

The application of both chemotherapy and targeted therapy has greatly improved the outcome of cancer patients, however, there is a large amount of evidence that indicates potential cardiotoxic complications of their use (1–3). Manifestation of the cardiotoxic effect of the administered drug can endanger the patient's life in two ways, both by direct impact on cardiac function and by the indicated discontinuation of the antineoplastic drug, which can worsen the prognosis of the oncology patient. Diagnosing cardiotoxicity is a difficult differential diagnostic task, because despite the well-known time correlation between receiving antineoplastic therapy and damage to heart function, the clinical picture of patients can be different. The cardiotoxic effect of specific therapy can be accompanied by minimal symptoms to severe AHF.

Fluoropyrimidines (5-fluorouracil and capecitabine) are antineoplastic drugs that have the most negative effect on the cardiovascular system, leading to anginal complaints such as stable angina pectoris, acute coronary syndrome (ACS) or the development of heart failure (4–6). Acute systolic heart failure due to 5-FU administration is rare unless associated with myocardial infarction, diffuse coronary vasospasm, toxic myocarditis, or Takotsubo cardiomyopathy (7). The most common adverse reaction caused by oxalipatin include nausea, vomiting, acute and cumulative peripheral sensory neuropathy and allergic reactions (8). As for cardiotoxicity, QT prolongation and ventricular arrhythmias have been reported after oxaliplatin (9), but direct toxic effect to the heart has rarely been described (10, 11). In order to prove the exclusive cardiotoxic effect of drugs as the cause of cardiac disease, in addition to the basic diagnostic methods [electrocardiogram (ECG), echocardiographic examination, and laboratory biomarkers of heart damage (brain natriuretic peptide- BNP; NT pro BNP)] for establishing a final diagnosis, it is recommended coronary angiography and cardiomagnetic resonance of the heart (CMR) (12). Not infrequently, intravascular ultrasound (IVUS) is performed which is an intravascular imaging modality primarily used in interventional cardiology to characterize lesion morphology, quantify plaque burden, guide stent sizing, assess stent expansion, and identify procedural complications. IVUS assessment can distinguish between calcified plaque, lipid, and neointimal proliferation.

According to the consensus of experts of the European Association of Cardiologists (ESC), Cancer therapeutics–related cardiac dysfunction (CTRCD) is defined as a decrease in the left ventricle ejection fraction (LVEF) of > 10 percentage points, to a value below the lower limit of normal (13). The presence of atherosclerotic plaques on angiography requires an assessment of their functional significance. Also, the possibility of existence an acute myocardial infarction (AMI) without obstruction of the blood vessels of the heart -MINOCA requires additional evaluation of the patient and the application of more sophisticated methods of proving myocardial damage. It is very important to differentiate these conditions in oncology patients, not only because of the application of different therapeutic modalities, but also because of the continuation of the treatment of the primary disease.

## Case report

A 52-year-old male, current heavy smoker, with no known comorbidities, was diagnosed with stage IV RAS wild-type adenocarcinoma of the rectum, with metastases in the liver and retroperitoneal lymph nodes in April 2022. About 2 weeks prior to the start of chemotherapy, he had been seen by a cardiologist due to asymptomatic ventricular extra systoles seen on routine ECG. At that time echocardiogram was unremarkable with LVEF of 65%; LV had normal end-diastolic diameter (EDD) and end-systolic diameter (ESD) (EDD/ESD−52 mm/33 mm). No disturbances were registered in segmental LV kinetics. LV diastolic function was preserved.

Upon admission to the Department of Oncology, he was started on FOLFOX-6 chemotherapy with planned administration of panitumumab, as per current guidelines (14). Panitumumab infusion was planned on day 3 of treatment, for technical reasons. He received 85 mg/m^2^ of oxaliplatin, followed by 400 mg/m^2^ leucovorin, 400 mg/m^2^ 5-FU i.v.bolus and 2400 mg/m^2^ continuous infusion of 5-FU over 46 h. Approximately 24 h into the continuous 5-FU infusion he started complaining of pain in the epigastrium that propagated toward the lower third of the sternum and slight nausea, with no shortness of breath, palpitations or dizziness. Infusion of 5-FU was stopped immediately. At this time, panitumumab had not yet been administered.

**Figure 1:**
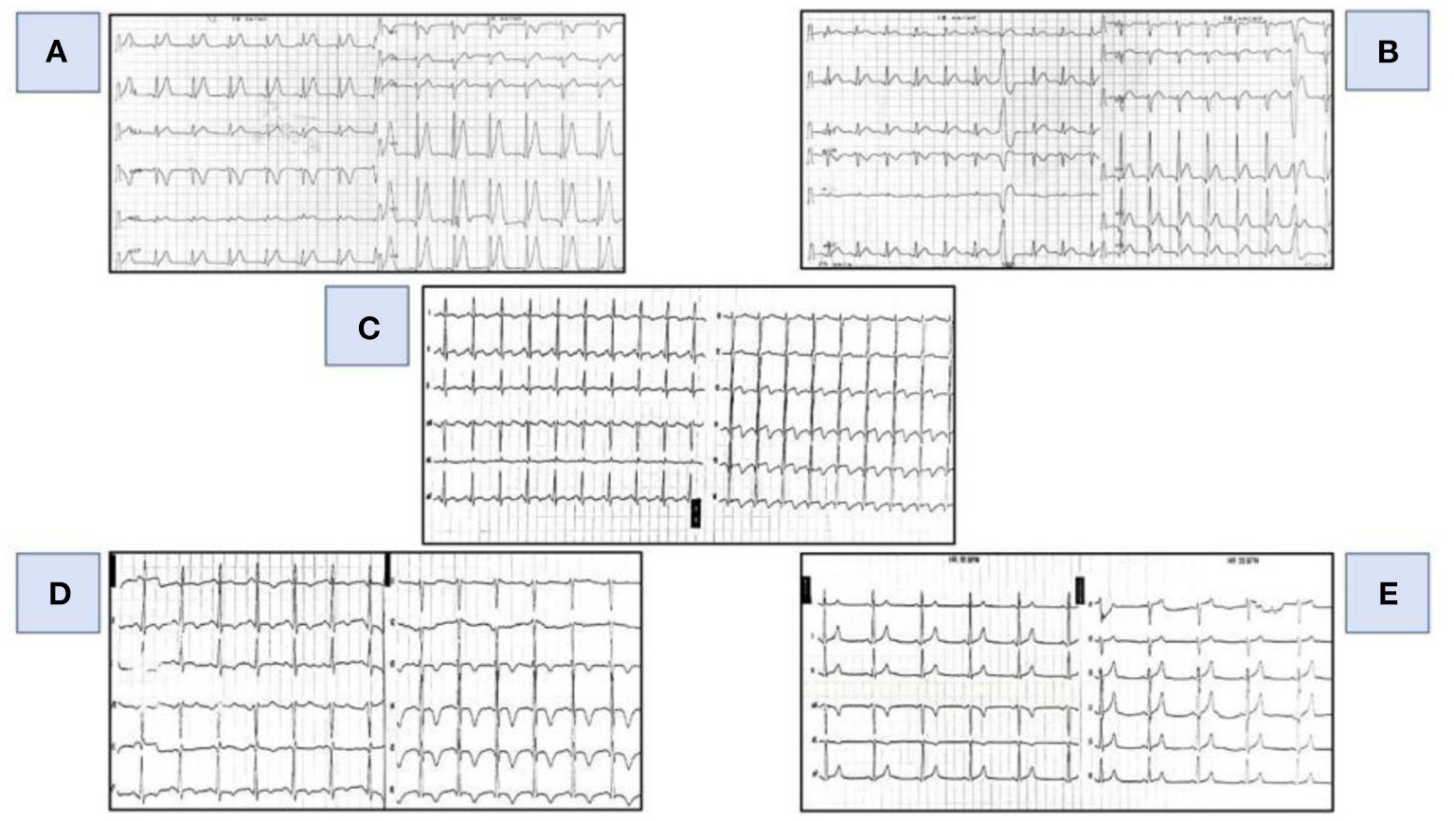
Evolutionary changes in the ECG. **(A)** (First day-07.14 AM: ECG at initial presentation): tall, spiky T waves diffusely in the ECG. **(B)** (First day-07.57 PM): concave ST elevation in the inferior and lateral series of leads. **(C)** (First day- 22.09 PM): ST elevation in the inferior and lateral series of leads with terminally negative T waves. **(D)** (Second day- 06.50 AM)**:** negative T waves in the inferior and lateral series of leads. **(E)** (Fifth day- 07.00 AM): normal electrocardiogram.

Upon examination he was hypertensive at 180/100 mmHg, slightly tachycardic with HR of 115/min, afebrile. ECG initially showed peaked T waves with no ST changes (Figure 1A). Initial values of high sensitivity Troponin (Hs-cTn) were in the reference range.

Due to persisting symptoms and evolution of ECG changes he was transferred to cardiac intensive care unit (ICU).

Upon admission to the ICU, the patient continued to complain of pain in the epigastrium spread to the lower third of the sternum, nausea and sweating. Vital signs on admission showed a heart rate of 95 beats/min, arterial blood pressure 110/80 mmHg on both arms, respiration rate 20/min, arterial oxygen saturation 96%. During the examination, the patient was conscious, oriented without focal neurological outbursts and cyanosis. Signs of heart failure (gallop rhythm—present third heart sound) and symptoms of heart failure (dyspnea, tachypnea, orthopnea) with the appearance of late-inspiratory crackles in the lower lung fields were registered.

In the ECG, after 10 h from the initial ECG, an evolution was registered in relation to the previous finding (concave elevation of the ST segment in the inferior and lateral series of leads with frequent single VES) (Figure 1B) and at 15 h from the initial ECG ST elevation is registered in the same series of leads with the appearance of terminally negative T waves (Figure 1C).

On the first therapeutic day, an ECHO of the heart was performed, where an enlarged LV was registered (EDD/ESD LV- 65/51 mm), with the presence of segmental wall motion abnormalities of the LV. Akinesia of the apical and medial third of the left ventricle as well as the basal segment of the interventricular septum was registered, while the remaining segments of the LV were hypokinetic. No signs of left ventricular apex ballooning were observed. Estimated EF using Simpson's Biplane method was 15–20%. There were no signs of valve disease or pericardial effusion. In laboratory analyses, non-significant increase in Hs-cTnT values was registered (12...168…64..12 ng/L; ref. range < 14 ng/L). A slight increase in Hs-cTnT was not accompanied by elevated values of creatinine kinase (CK) (64...40...20 U/L; ref. range 0.0-200 U/L), MB fraction of CK (CK-MB) (2 U/ L; ref. range < 25 U/L) nor lactate dehydrogenase (LDH) [340...328 (ref. value 220-460 U/L)]. Although we knew that Hs-cTnt can be easily elevated in patients with AHF, such as our patient, we suspected that it was an acute coronary syndrome. There was no increase in inflammatory markers (CRP, leukocytes and procalcitonin) as well as nitrogen substances and D-dimer.

In view of the clinical picture, ECG changes as well as the findings of echocardiography, the patient underwent cardiac catheterization, which did not register angiographically significant narrowing of the large blood vessels of the heart. Coronary artery vasospasm was not visualized during coronary angiography. Considering the findings of the coronary angiography, a working diagnosis of myocardial infarction without obstruction of the blood vessels of the heart was made—MINOCA (15).

**Figure 2:**
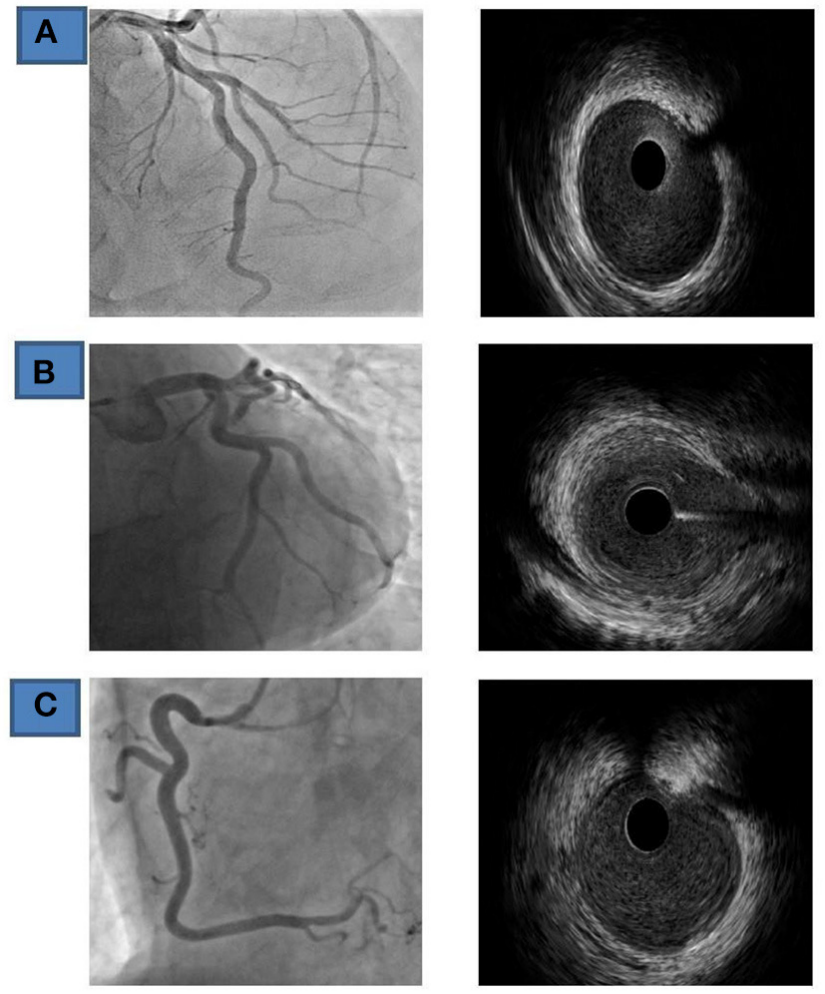
Coronary findings and IVUS (Fifth day of admission) **(A)** LAD, left anterior-descending coronary artery. **(B)** Cx, circumflex coronary artery; **(C)** RCA- right coronary artery.

In order to establish/exclude the diagnosis, the patient underwent in the second act automated intravascular ultrasound (IVUS) system, pull/back interrogation (0.5 mm/s) of all three coronary arteries. IVUS showed normal trilaminar appearance of vessel wall and absence of atherosclerotic disease. In addition, signs of plaque rupture, plaque erosion or thrombus were not found in either of all three coronary arteries (Figure 2).

Medical treatment continued (coronary vasodilators, calcium channel blockers, ACE- inhibitors, beta-blockers, low-molecular-weight heparin, diuretics with the use of antiarrhythmic—amiodarone) in the ICU, where he was monitored all the time. Rapid evolutionary changes on daily basis were still registered in the ECG, and on the 2nd day of therapy negative T waves were registered in the inferior and lateral leads (Figure 1D) while, on the 5th day of therapy, a normal ECG was registered (Figure 1E).

During the next 7 days, control echocardiographic examinations were performed on several occasions. Given that we still did not have a clear diagnosis, in order to eliminate the differential diagnostic dilemma between myocardial infarction without changes in the blood vessels of the heart (MINOCA) and toxic cardiomyopathy caused by 5-FU, the patient underwent CMR. It was performed on the seventh therapeutic day from the onset of symptoms according to ESC recommendations (16). Cardio magnetic resonance should have considered the presence of scar in the endocardium/myocardium, the presence of micro- vascular obstructions, the presence of hemorrhage in the myocardium, myocardial edema and the detection of the so-called “gray zone”—the zone at risk or to prove signs of the presence of dilated cardiomyopathy caused by 5-FU.

Examination of the heart was performed in standard planes using functional True FISP sequences and TSE morphological sequences in three planes without contrast medium application, as well as after contrast application with IR sequence and T1 and T2 maps using Modified Look Locker inversion recovery (MOLLI) sequence. Reduced LV systolic function was registered (EF 40%); LV was dilated (EDD/ESD- 62/47 mm), normal wall thickness, enlarged EDV (202 ml; ref 77–195 ml) and ESV (121 ml; ref 19–72 ml), overall hypo contractile without segmental LV wall abnormalities.

**Figure 3:**
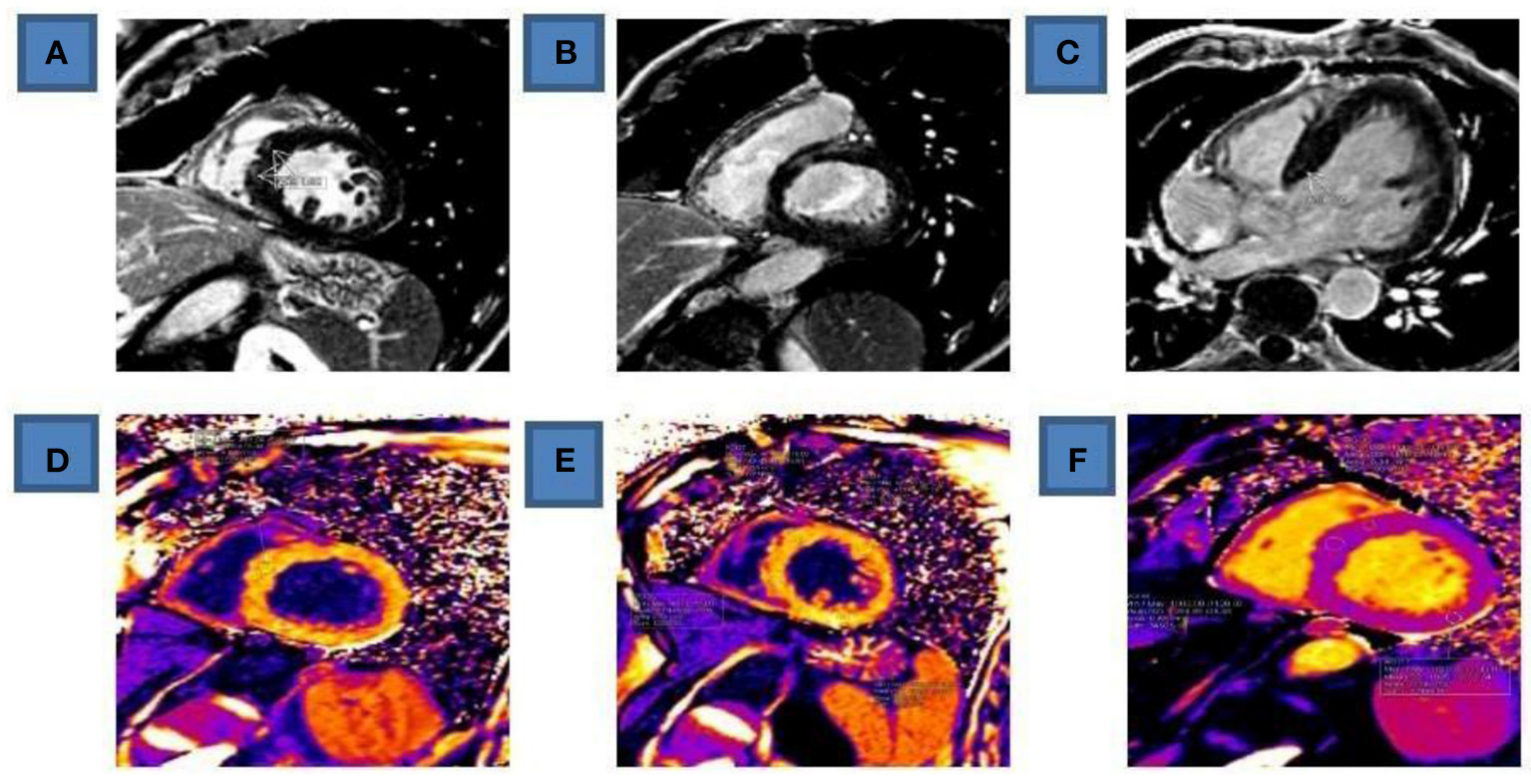
CMR of the heart (Fifth day of admission). **(A)** Postcontrast LGE in medial septum on PSIR sequence, SAX. (**B)** Basal anteroseptal postcontrast LGE on PSIR sequence, SAX. **(C)** Basal septum LGE phenomenon as well as pericard LGE in basal and medial lateral wall on PSIR sequence, four chamber view. **(D)** Pathological postcontrast T1 mapping (values > 500 ms) in basal septum, on MOLLI sequence, SAX. (**E)** Pathological postcontrast T1 mapping (values > 500 ms) in medial segments of all walls, circumferentially, on MOLLI sequence, SAX. **(F)** Pathological precontrast T1 mapping (values > 1100 ms) in basal septum and lateral wall. LGE, Postcontrast late gadolinium enhancement; PSIR, Phase sensitive inversion recovery; SAX, short axis view; MOLLI, Modified Look Locker inversion recovery.

Post-contrast, a smaller linear zone of late gadolinium accumulation (LGE) was observed—septal fibrosis intramyocardially in the basal part of the septum with inhomogeneous opacification of the entire myocardium, primarily as part of the post-therapeutically altered myocardium (cardio toxicity). No signs of localized edema or necrosis of LV were registered. According to radiologists and CMR findings, changes in the myocardium first correspond to changes in cardiotoxicity (Figure 3).

During hospitalization, a drop in TnT was registered in the laboratory with the normalization of natriuretic peptides.

Transthoracic echocardiography (TTE) on the fifth day of admission revealed an enlarged LV (EDV 182 ml, ESV 113 ml), severely hypokinetic, with depressed EF (30%) and spontaneous contrast within the cavity. Global longitudinal function was significantly reduced (GLS-9.5%) with prominent mechanical dispersion (PSD 80.5 ms), detected by 2D speckle tracking echocardiography.

**Figure 4:**
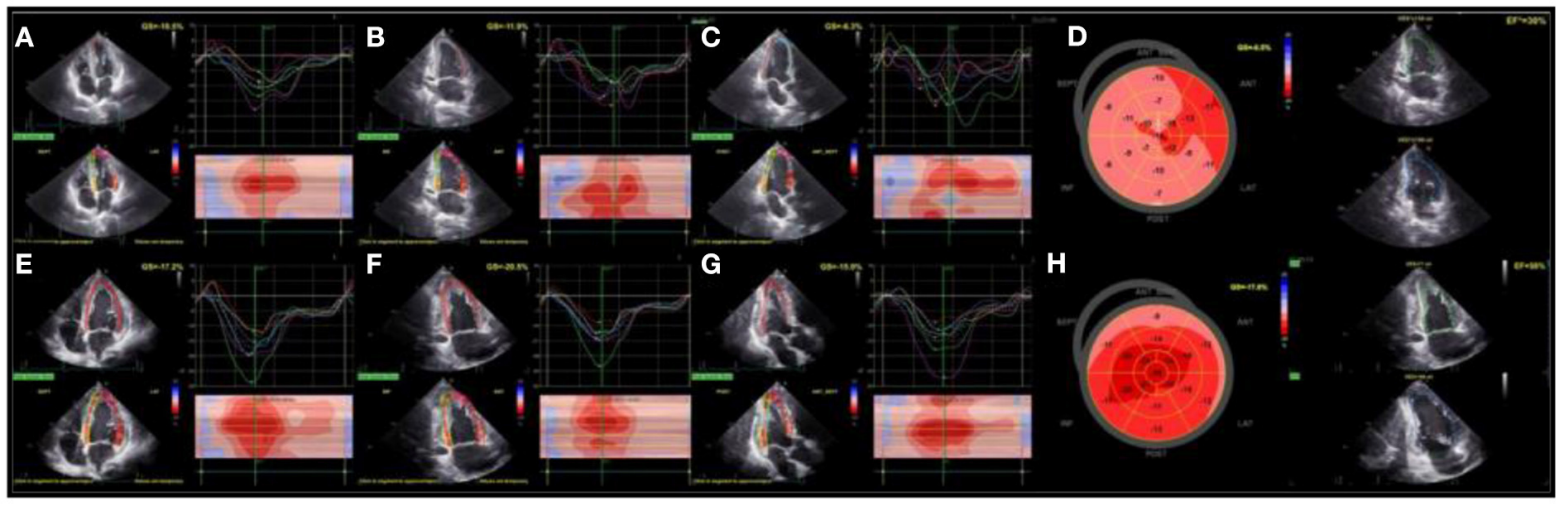
Assessment of LV function with TTE (On the fifth day of admission): LV systolic function was severely impaired, including impaired and asynergic longitudinal deformations that can be appriciated from speckle tracking analysis of apical 4- chamber **(A)**, 2-chamber **(B)**, and 3-chamber **(C)** views and globally from polar map **(D)**. During the follow-up (eighteenth day), LV EF and longitudinal deformations significantly improved **(E–H)**. LV, left ventricle; TTE, transthoracic echocardiography; EF, left ventricular ejection fraction.

Thirteen days after, TTE confirmed the presence of enlarged LV (EDV 168 ml, ESV 71 ml), but with significantly better LV EF (58%), improved global longitudinal function (−17.6%), and significantly more synergic intraventricular contractions (PSD 44.6 ms) (Figure 4).

The patient was discharged home in good general condition. Frequent check-ups by a cardiologist are advised. Further oncological treatment was advised based on the opinion of the cardiology-oncology council (Supplementary Figure 1).

## Discussion

Thanks to the recommendations of the ESC and the American Heart Association (AHA), it would be logical that the diagnosis of AMI based on the clinical picture of the patient, ECG changes and elevated troponin values is very easy (16–18). However, we are faced with the fact that we also have a certain number of patients in whom the diagnosis is difficult or in whom the diagnosis of AMI is incorrectly established. According to a systematic review by Kwok, Chun Shing et al., which included 15 studies, it was shown that the diagnosis of AMI is missed in 1–2% of patients (19).

Reasons for missed AMI diagnosis include incorrect electrocardiogram interpretation and failure to order appropriate diagnostic tests. Navi et al. showed that patients with cancer have a 3-fold higher risk of AMI compared to patients without cancer (20). The prothrombogenic state and hyper viscosity of blood in patients with cancer can lead to the formation of arterial thrombosis, while the use of drugs as part of chemotherapy can lead to endothelial cell damage predisposing to erosion and rupture of the atherosclerotic plaque and thus lead to AMI type I. On the other hand, AMI Type II can be provoked by tachycardia, hypotension, hypoxia or anemia, as well as vasospasm due to the use of chemotherapy drugs (21).

In our patient, the presence of pain in the epigastrium with propagation in the lower third of the sternum, changes in the ECG and new changes in the echocardiography, which he did not have before chemotherapy, as well as slightly elevated values of Hs-cTnT raised suspicion that it is AMI. However, elevated values of Hs-cTnT in AHF may be due to non-ischemic events (e.g., increased afterload, increased preload, oxidative stress, etc.) (22). Studies have shown that in patients with AHF, a certain pathological stimulus can cause the release of troponin directly from the cytosol of otherwise intact myocytes, which are called the cytosolic pool (22). Also, the effects of stretching, increased volume, and pressure overload that occur with AHF should not be overlooked. Numerous stressors, such as inflammatory mediators and neurohumoral stimulation may also have an effect on increasing cTn values in patients with AHF. In the ADHERE study, 75% of patients hospitalized with AHF (67 924) had detectable levels of cTn (cTnI >0.4 ng/ml or cTnT >0.01 μg/l). When a higher threshold for cTn values was used (cTnI of 1.0 ng/ml or cTnT of 0.1 g/l), about 6.5% of patients with AHF had values above this level (23). Similar data were shown by the study of Logeart D. and colleagues in a patient with heart failure of non-ischemic etiology (24). In the study by You JJ and colleagues in which 2025 AHF patients were analyzed, the prevalence of cTnI values above the 99th percentile was registered in 34.5% of patients (25). Increased troponin values can be detected early in chemotherapy administration, long before LV functional damage is detected by available techniques. Cardinale et al. by serial measurement of cTnI levels in serum during the administration of chemotherapy showed that elevated TnI values are associated with a progressive decrease in LVEF (26).

In relation to AMI with obstruction of blood vessels (> 50% obstruction), myocardial infarction with non-obstructive coronary arteries (MINOCA) is a heterogeneous clinical entity, characterized by clinical evidence of myocardial infarction (MI) with non-obstructive coronary arteries on angiography (≤ 50% stenosis) (27). The prevalence of MINOCA is 3.5–15% (27). When MINOCA is suspected in most cases, additional invasive and non-invasive tests are needed to establish its diagnosis. IVUS often diagnoses the presence of plaque rupture or ulceration, which is a frequently missed finding in women who have had MINOCA (28). The presence of plaque rupture or ulceration in the coronary arteries in patients with MINOCA is diagnosed with IVUS in about 40% of cases (29, 30). Based on the aforementioned research, we first performed IVUS and then CMR in our patient. ESC recommendations suggest the use of CMR in all patients with suspected MINOCA, as it identifies its underlying cause in 87% of patients (17). In our patient, the application of CMR helped to resolve this differential- diagnostic dilemma.

The occurrence of cardiac dysfunction, depending on the manifestations of the disease after the administration of chemotherapy, can be acute, subacute and chronic (31). Changes in cardiac function can be reversible or irreversible (32). Our patient had acute cardiac dysfunction that was reversible. AHF, cardiogenic shock, and even sudden cardiac death if not explained by coronary vasospasm can be caused by myocardial inflammation or the presence of Takotsubo cardiomyopathy as a consequence of the action of 5-FU (33–36).

Anthracyclines are a group of drugs that most often lead to cardiotoxicity (AHF occurs in 2–4% of patients), followed by 5-FU pyrimidine analog, which is widely used for the treatment of many solid tumors, including colorectal, breast and head and neck cancers (33, 37). Incidence of cardio toxicity after application 5-FU moves from 1 to 35% in various studies and mortality from 2 to 13% (38–41). The incidence of the development of myocardial damage depends on the administered dose, the distribution of the drug and the method of its administration. The greatest risk of developing cardiotoxicity is during the first administration of the drug (33, 40). However, there is a schedule-dependent difference, with a higher risk of developing cardiac toxicity when using continuous infusion of 5-FU in comparison to bolus infusion, possibly related to the short half-life of 5-FU, which is 15–20 min (39, 42).

The most frequently documented symptom of cardiotoxicity with 5-FU administration is chest pain. However, care should be taken in patients with cancer because the symptoms of AMI can be atypical, with some authors stating that dyspnea (and not chest pain) is the most common presentation of the disease (43). In a study by Jensen et al., during treatment of colorectal cancer patients with the FOLFOX regimen, the incidence of chest pain was found to be about 8.5% (44). In our patient, the cardio toxicity of 5-FU manifested itself during the continuous infusion of 5-FU and was accompanied by pain in epigastrium with propagation in the lower third of the sternum. In patients treated with 5-FU, anginal complaints occur in 45% of patients, and heart failure in 2% of patients (45). Oxaliplatin, unlike 5-FU, more often leads to gastrointestinal complaints, hypersensitivity reactions, even anaphylactic shock (46) and far less often has cardiac side effects in the form of disturbances in the electrical activity of the heart, which can be manifested by the appearance of arrhythmias or conduction blocks (9, 47).

A marked heterogeneity has been shown in terms of ECG changes in patients treated with 5-FU (48–50). Changes in the ECG can easily mislead physicians when it comes to cardio toxicity with 5-FU. Ischemic changes in the ECG are found far more often in patients with previously proven coronary disease (51). Changes in the form of hyper acute T waves have also been described (52). Mizuno et al. reported a case of 5-FU-induced cardiomyopathy, which presented with chest pain, ECG-changes in the form of diffuse ST-elevation, diffuse LV kinetic disturbances with normal coronary arteries on angiography. The aforementioned authors considered that coronary vasospasm was not the cause of cardiomyopathy in this patient (53). The changes in the electrocardiogram in our patient could indicate a possible pericardial affection caused by the toxic effect of the drugs. It is recommended that all patients who receive 5-FU and develop chest pain with ischemic changes in the ECG undergo angiography. Most often, the findings are normal, and transient changes in the form of ST elevation are explained by transient vasospasm, which is registered angiographically (7, 54).

In some cohorts, coronary artery vasospasm was directly visualized during coronary angiography, but this was not the case in our patient. Although no vasospasms were registered during coronary angiography, shorter vasospasms could not be ruled out. Our patient could not have diffuse vasospasms because their occurrence would be followed on CMR by myocardial edema and late accumulation of gadolinium in the subendocardial part of the myocardium. Even the application of nitroglycerin and calcium blockers in our patient did not lead to the resolution of changes in the ST segment, which would otherwise happen if it were really about vasospasm. Various studies have shown different coronary angiography findings, from clear vessels to the presence of significant occlusions (39, 55, 56). In our patient, IVUS did not show the presence of plaque rupture, plaque erosion, thrombosis, i.e., significant stenoses on the blood vessels of the heart.

Sudden HF in our patient is not a consequence of Takotsubo cardiomyopathy, not only because of the absence of echocardiographic criteria (LV ballooning), but also because the Gothenburg criteria exclude the diagnosis of Takotsubo syndrome if there is suspicion of drug-induced cardiotoxicity (57). Nor were the 2008 Mayo criteria for the diagnosis of Takotsubo cardiomyopathy met (58). The use of CMR excluded the existence of segmental abnormalities in kinetics, myocardial edema (which would be in favor of toxic myocarditis) as well as microvascular obstructions, all of which were in favor of acute dilated toxic cardiomyopathy. We also consider it almost impossible that LV changes caused by acute myocarditis and consequent severe dilated cardiomyopathy on echocardiography are completely reversible and that in such a short period of time as in our patient. In patients with myocarditis and severely impaired cardiac function (< 25%) who were treated with immunomodulatory drugs, it was shown that the recovery of cardiac function usually occurs between 6 and 14 weeks after the start of therapy. In about a third of patients, that recovery begins only between the 2nd and 4th months (59). In acute myocarditis, the left ventricle is damaged for a long time with reduced systolic function. A possible pathophysiological cause of acute toxic impairment of LV function after 5-FU administration could be a sudden decrease in the level of adenosine triphosphate, which is also described in the literature (7, 60).

## Conclusions

In addition to the very great therapeutic effect of 5-FU in the treatment of patients with rectal cancer, its use is often associated with heart diseases. Fluoropirimidines are amongst the most commonly-used cardiotoxic antineoplastic drugs, with cardiotoxic effects ranging from asymptomatic ECG changes to sudden cardiac death, suspected to be caused by coronary vasospasm. Although the literature states that coronary vasospasms, thrombosis and toxic myocarditis are the most common causes of AHF LV in patients treated with 5-FU, it can often occur due to the direct toxic effect of the drug. Our case report presents an uncommon clinical manifestation of 5FU cardiotoxicity. Summarizing the knowledge available in the world literature so far, we have differentially considered all cardiac diseases that can occur due to the toxic effect of 5-FU. If rapid reversibility of changes on the electrocardiogram and rapid echocardiographic recovery of heart function is registered, this would be in favor of the fact that the disease is not of ischemic origin. Newly diagnosed acute dilated cardiomyopathy followed by heart failure in the absence of angiographically significant stenosis on the blood vessels of the heart and CMR criteria for ischemic or inflammatory myocardial disease should always raise the suspicion of toxic reversible cardiomyopathy caused by 5-FU. In the case of our patient, the most modern diagnostic methods were used to rule out differential-diagnostic dilemmas and establish a diagnosis. Taught by our experience, we recommend a mandatory ECHO strain for every patient before chemotherapy, and if there are cardiac complications, during or after the application of chemotherapy, a serious cardiological-oncological approach, both in the administration of an interventional therapeutic regimen and in order to continue the adequate treatment of an oncological patient.The fact that the mechanisms by which 5- FU leads to myocardial damage are still not fully explained requires the implementation of new clinical studies that will resolve this dilemma.

## Data availability statement

The original contributions presented in the study are included in the article/Supplementary material, further inquiries can be directed to the corresponding author.

## Author contributions

Conceptualization, methodology, investigation, data curation, writing, and original draft preparation: RL and LD. Original draft preparation: JS and MA. Investigation: DT-Z, DO, and ON-A. All authors have read and agreed to the published version of the manuscript.

## Conflict of interest

The authors declare that the research was conducted in the absence of any commercial or financial relationships that could be construed as a potential conflict of interest.

## Publisher's note

All claims expressed in this article are solely those of the authors and do not necessarily represent those of their affiliated organizations, or those of the publisher, the editors and the reviewers. Any product that may be evaluated in this article, or claim that may be made by its manufacturer, is not guaranteed or endorsed by the publisher.

## Supplementary material

Supplementary Figure 1 Values of laboratory parameters and results of diagnostic methods in relation to the time period of hospitalization.
