# Supplementary figures and images for: Case report: Acute toxic myocardial damage caused by 5-fluorouracil—from enigma to success
Source: Front Cardiovasc Med. 2022 Oct 18;9:991886. doi: 10.3389/fcvm.2022.991886 (PMC9622946; doi:10.3389/fcvm.2022.991886)

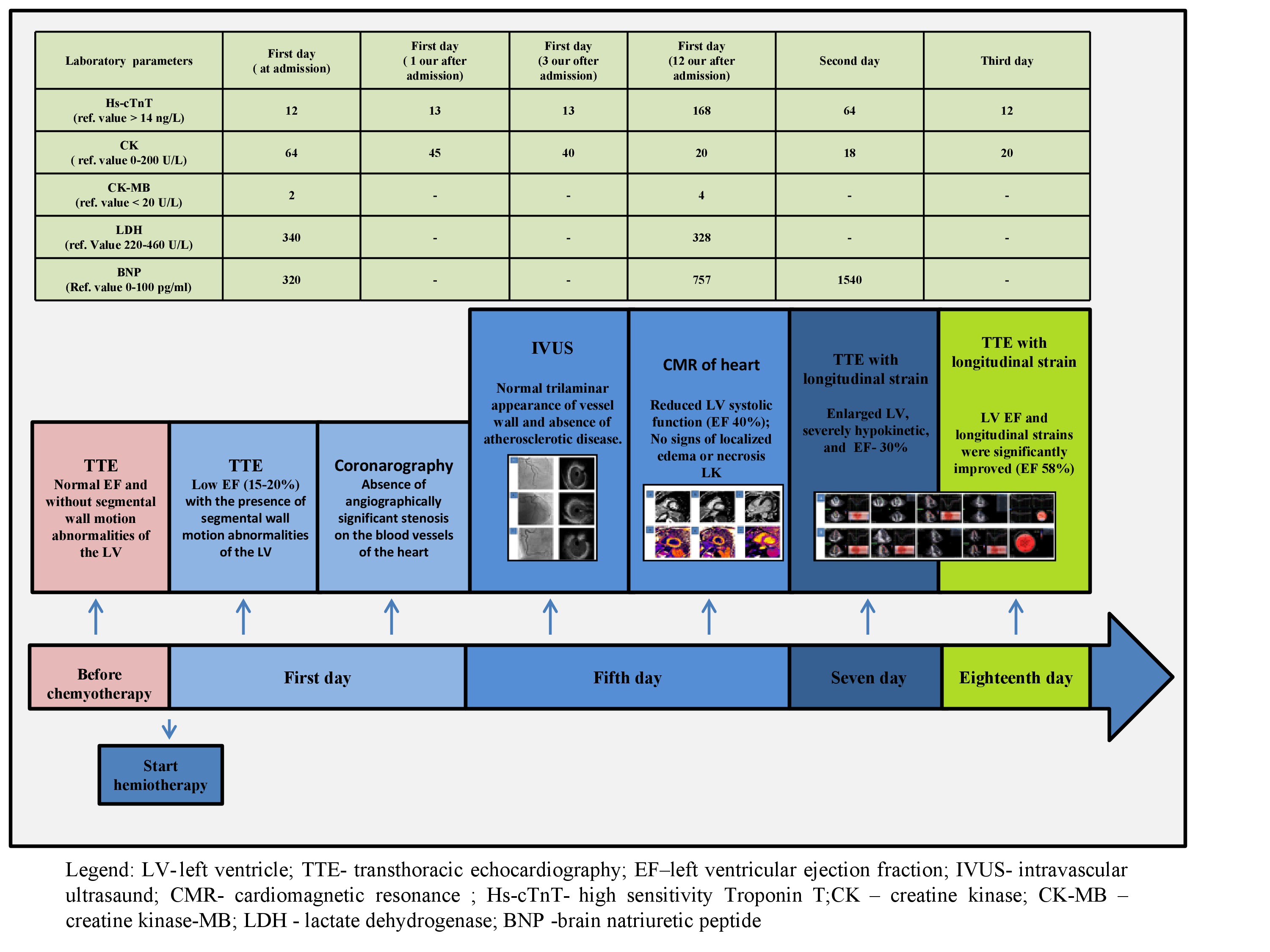

Supplement: Supplementary Figure 1 — Values of laboratory parameters and results of diagnostic methods in relation to the time period of hospitalization. [file Image_1.JPEG]
